# Supplementary material for: Neural Representations Behind ‘Social Norm’ Inferences In Humans
Source: Sci Rep. 2018 Aug 28;8:12943. doi: 10.1038/s41598-018-31260-5 (PMC6113313; doi:10.1038/s41598-018-31260-5)
Supplement: Supplementary file 1 — Supplementary Figure 1 [file 41598_2018_31260_MOESM1_ESM.pdf]

Title: Neural Representations Behind 'Social Norm' Inferences In Humans

(Supplementary Information)

Authors: Felipe Pegado*[1,2,3], Michelle H.A. Hendriks[1,3], Steffie Amelynck[1], Nicky Daniels[1], Jessica Bulthé[1], Haemy Lee Masson[1], Bart Boets[2,3], Hans Op de Beeck*[1]

Affiliations:
Department of Brain and Cognition, KU Leuven, 3000 Leuven, Belgium
Center for Developmental Psychiatry, Department of Neurosciences, KU Leuven, 3000 Leuven, Belgium
Leuven Autism Research consortium, KU Leuven, 3000 Leuven, Belgium

Content: 1 supplementary figure (S1)

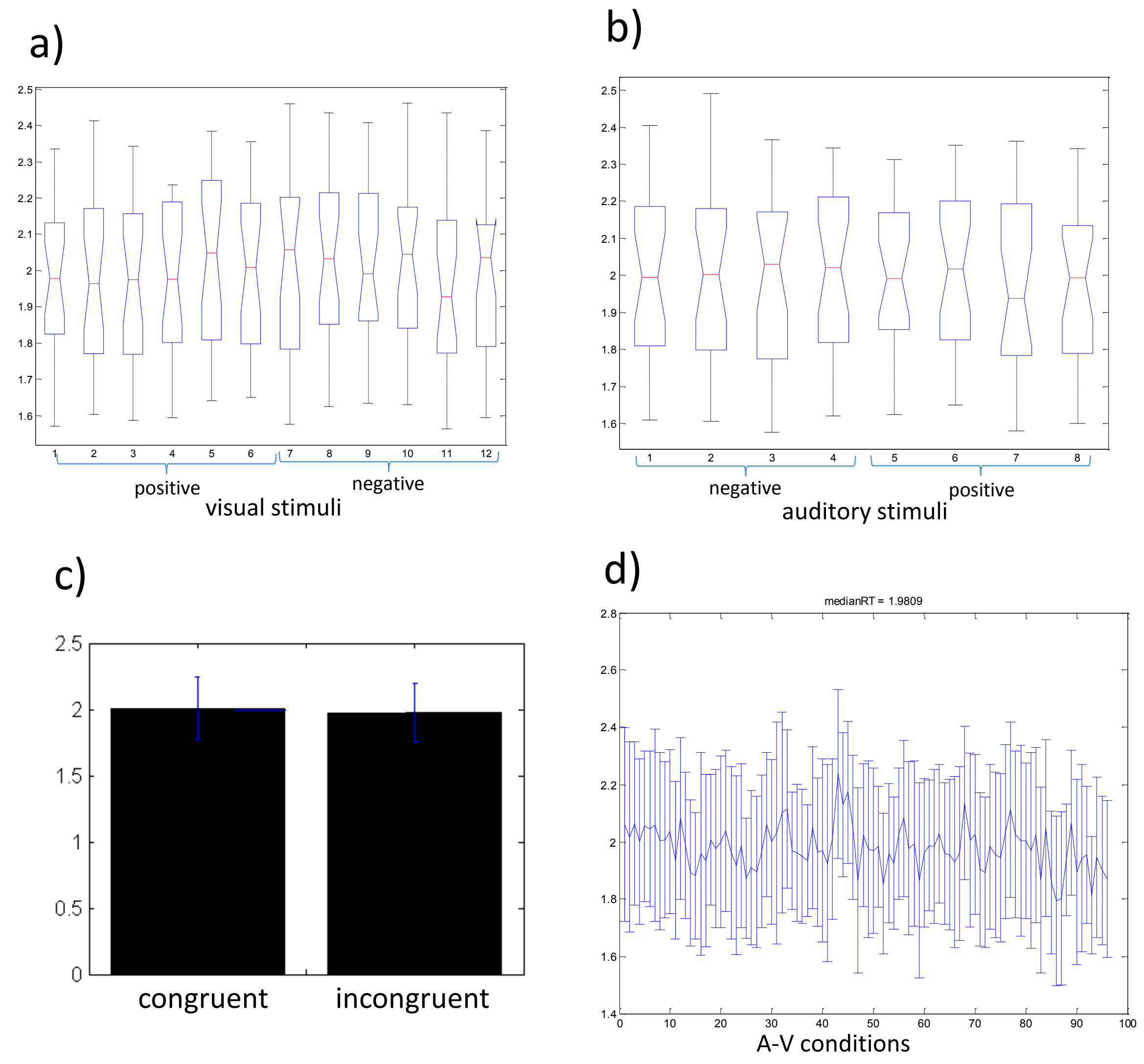

Supplementary Figure S1. **Median Response Times (RTs) in seconds**. **a**) Boxplots of RTs as a function of the visual stimuli (first six are positive and last six are negative valence images). **b**) Boxplot of RTs as a function of the auditory stimuli (first four are negative and last four are positive valence vocalizations). **c**) barplots for congruent and incongruent trials. **d**) RTs for each of the 96 individual audio-visual (A-V) combination conditions. For A and B, on each box, the central mark indicates the median, and the bottom and top edges of the box indicate the 25th and 75th percentiles, respectively. The whiskers extend to the most extreme data points. For C and D, the Error bars represent +/- 1 SD.
